# Architecture and regulation of nanoscale chromatin domains

**DOI:** 10.1038/s41467-026-71213-5

**Published:** 2026-04-01

**Authors:** Vinayak Vinayak, Melike Lakadamyali, Vivek B. Shenoy

**Affiliations:** 1 Center for Engineering Mechanobiology, University of Pennsylvania, Philadelphia, PA USA; 2 Department of Materials Science and Engineering, University of Pennsylvania, Philadelphia, PA USA; 3 Department of Physiology, Perelman School of Medicine, University of Pennsylvania, Philadelphia, PA USA

**Keywords:** Computational biophysics, Biological physics, Super-resolution microscopy, Epigenetics

## Abstract

Nanoscale chromatin domains have emerged as fundamental units of mammalian genome organization during interphase and mitosis. Single-molecule localization microscopy now enables their direct visualization, revealing conserved features including characteristic packing, enrichment of linker histones, and radial stratification of histone marks. These domains act as dynamic regulators of gene activity, remodel in response to developmental and environmental cues, and become disrupted in disease. Experimental findings and biophysical modelling point to internucleosomal interactions and epigenetic reactions as key drivers of their organization. By situating them alongside lamin- and nucleolus-associated domains, we propose a unified biophysical framework for genome organization across scales. Their recurrent disruption in aging and disease makes them compelling targets for diagnosis and intervention.

## Abstract (web summary)

Conserved 50-200 nm chromatin domains add a new layer to genome organization beyond loops. This Review integrates imaging and biophysics to show how epigenetic activity and transcription shape these domains to regulate cell identity.

## Introduction

The human genome is compacted several thousand-fold to fit within the nucleus, yet remains accessible to enable precise and dynamic gene regulation. How this remarkable balance between compaction and function is achieved remains a central question in cell biology. The spatial organization of chromatin within the nucleus is therefore fundamental to genome regulation, yet still only partially understood. At the broadest scale, chromatin segregates into euchromatin and heterochromatin: two chromatin states distinguished by transcriptional activity, accessibility, and histone modifications. Euchromatin, characterized by high DNA accessibility and active marks such as H3K9ac and H3K27ac, supports gene expression, while compact, inaccessible, transcriptionally silent heterochromatin is enriched in repressive marks like H3K9me3 and H3K27me3 along with associations with proteins such as HP1^1–5^. This binary framework has historically underpinned models of genome function, yet recent imaging and sequencing studies reveal additional layers of chromatin organization beyond this simple dichotomy.

Over the past decade, genome-wide chromosome conformation capture techniques (such as Hi-C^6^ and Micro-C^7^), together with advances in fluorescence-based microscopy, have revealed hierarchical layers of three-dimensional genome folding. These include chromosome territories, A/B compartments, topologically associating domains (TADs), and chromatin loops, where the majority of loops are formed and stabilized by architectural proteins such as CTCF and cohesin. Concurrently, physics-based models have successfully explained the occurrence of these scales of organization through phase separation and the action of active motors^8,9^. Altogether, these features, often derived from ensemble measurements, have transformed our understanding of how multiscale genome topology interacts with and affects transcriptional control and cell phenotype.

However, these refined models remain incomplete, constrained by diffraction-limited microscopy and the still-developing reliability of single-cell sequencing approaches. More recently, super-resolution imaging approaches, including single-molecule localization microscopy (SMLM)^10^, structured illumination microscopy (SIM)^11,12^, and stimulated emission depletion (STED)^13,14^ microscopy, have overcome the classical diffraction limit and enabled direct visualization of nanoscale chromatin organization. More specifically, STORM^15^ imaging approaches have uncovered a striking layer of nanoscale chromatin organization: dense, spatially discrete, heterogeneous domains ranging from ~50 to 200 nanometers^16,17^. These nanoscale structures, consistently observed across mammalian cell types, display conserved biophysical and epigenetic characteristics that lend them distinct architectural features. Notably, they persist even in the absence of classical loop extrusion machinery, highlighting a distinct mechanistic foundation. Their dynamic reorganization during development and disease further underscores their potential role in cell fate decisions. Yet, existing physics-based models only partially explain their emergence, suggesting that these domains may represent a privileged layer of genome regulation that complements established higher-order structures.

In this Review, we synthesize emerging evidence on nanoscale chromatin domains. We use the term ‘chromatin nanodomains’ or ‘nanoscale chromatin domains’ to encompass the 50-200 nm clusters variously described as nucleosome clutches, chromatin domains or packing domains. Rather than introducing new nomenclature, we aim to unify these observations within a coherent biophysical framework. We examine the imaging advances that enabled their discovery, their physical and epigenetic architecture, and their interactions with transcriptional machinery and environmental cues, including implications for disease and epigenetic therapy. By integrating these insights across scales, we position chromatin nanodomains as a fundamental and previously underappreciated layer of genome organization and outline the key conceptual and technical challenges ahead.

## Super-resolution imaging to visualize chromatin domains

**Fig. 1:**
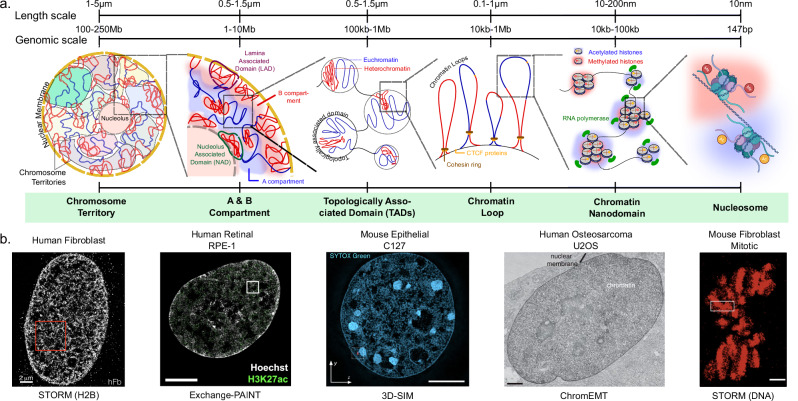
Positioning nanoscale chromatin domains within multiscale genome organization. **a** Genome organization spans a hierarchy of spatial and functional domains, from whole chromosome territories to individual nucleosomes. The schematic illustrates the key architectural levels (chromosome territories, compartments, TADs, loops, nanodomains, and nucleosomes) alongside their characteristic spatial scales. Chromatin nanodomains (~50–200 nm) represent a recently uncovered layer of organization, enriched in specific histone modifications and transcriptional regulators, yet invisible to diffraction-limited microscopy. This schematic illustrates approximate length scales of chromatin organization across the continuum. The cartoon is not drawn to nucleosome number and does not imply a strict hierarchy. Sizes of TADs (typically a few hundred nanometers to micrometers) and loops (from tens to hundreds of nanometers) along with nanoscopic chromatin domains reflect broad ranges and are shown only as conceptual models. The depiction of “nanodomains” as clusters of a few nucleosomes is diagrammatic and does not represent their actual nucleosome count (which spans tens to hundreds depending on genomic span and compaction). **b** Their discovery has been enabled by super-resolution fluorescence microscopy and electron-based methods. Imaging panels demonstrate how diverse techniques converge to resolve nanoscale domains, collectively establishing their conserved and distinct structural identity^16,20,23,24,26^ Created in BioRender. Vinayak, V. (2026) https://BioRender.com/8imdt2d.

Building on recent advances in genome-wide conformation capture^18^ and the conceptual framework of euchromatin-heterochromatin segregation, super-resolution imaging has uncovered a deeper stratum of chromatin architecture composed of nanoscale, majorly sub-diffraction-limited domains ranging from ~50 to 200 nm^16^. Historically, chromatin was widely thought to fold into a canonical 30 nm fiber; however, this model has been largely overturned by super-resolution and electron microscopy studies, which revealed irregularly packed, flexible chains of nucleosomes with no consistent higher-order fiber^16,19**–**21^. This conceptual shift opened the door to investigating chromatin organization beyond diffraction-limited resolution. A convergence of imaging techniques, including super-resolution fluorescence microscopy, correlative electron imaging, and multiplexed genomic FISH, has revealed that these domains are conserved across mammalian cell types (Table 1 and Fig. 1). Their robust spatial characteristics and independence from topological constraints of looping machinery suggest they represent a mechanistically distinct level of genome architecture that complements established models of multiscale nuclear organization.

**Table 1:** Summarizing the key imaging tools to observe nanoscale domains, highlighting their advantages, limitations, and the insights they offer^5,16,20,22,23,25,26,42^

Technique	Resolution	Labeling strategy	Strengths	Limitations	Key contributions
STORM (Ricci et al., Xu et al.)	~20–30 nm	Antibodies (e.g., H2B, PTMs)	Revealed nanoclusters and LAD morphologies	Requires fixation; No gene level specificity	Defined nucleosome clutches; histone mark stratification
PALM (Nozaki et al.)	~30–50 nm (live)	Photoactivatable fluorescent proteins	Live-cell dynamics; identifies coherent motion	Lower spatial resolution; specialized analysis	Visualized dynamic chromatin domains in live cells
3D-SIM + FIB-SEM (Miron et al.)	3D-SIM: ~100 nm (~300 nm axial) FIB SIM: ~10 nm (voxel ~4 nm)	DNA dyes + EM stains	Correlative resolution; identifies structural zonation	FIB-SEM: Limited molecular specificity	Mapped CD chains and spatial zonation at domain surfaces
ChromEMT (Ou et al.)	~8 nm	DNA-binding photooxidizing dye	High-resolution ultrastructure of chromatin	EM-based; fixed cells only	Rejected 30-nm fiber; confirmed disordered chromatin chains
ChromSTEM (Virk et al.)	~2–4 nm	Electron density contrast	Density quantification; detects fractal packing	No molecular specificity	Determined fractal dimension; cohesion-independent domains
Oligopaint-STORM (Boettiger et al.)	~30 nm	Locus-specific DNA probes	Folding rules by epigenetic state	Complex probe design; fixed cells	Showed scaling differences by chromatin state
PWS (Virk et al.)	~20–200 nm (ensemble)	Label-free	Live-cell; chromatin packing scaling	No gene-level specificity	Linked chromatin structure to transcriptional plasticity
3D-SIM + Oligopaint FISH (Szabo et al.)	~100 nm	DNA locus-specific probes	Direct visualization of TADs and nanodomains in single cells; cohesin/CTCF perturbation tested	Fixed cells only; requires well-designed probes	Revealed nanodomains as structural units persisting without loop extrusion components
Exchange-PAINT + HIST (Rahman et al.)	~22 nm lateral/~55 nm axial	Oligo-conjugated antibodies and DNA	High multiplexing; 3D nanodomain profiling of proteins, PTMs, and DNA	Requires fixation; complex analysis	Revealed nanoscale spatial transitions of nuclear proteins below ~300 nm

### Super-resolution fluorescence imaging reveals heterogeneous nanodomains

Initial insight came from Stochastic Optical Reconstruction Microscopy (STORM) imaging of histone H2B in mammalian nuclei by Ricci et al., who showed that nucleosomes organize into heterogeneous, nanoscale clusters or “clutches” varying in size and density depending on cell pluripotency state^14,16^. Larger and denser clutches were associated with heterochromatin and excluded RNA polymerase II (RNA polII), suggesting that they have lower transcriptional activity, while smaller, more dispersed clutches colocalized with RNA polII, suggesting active transcription sites. Simultaneously, the discovery of the domains was confirmed by Prakash et al., who used super-resolution localization microscopy on meiotic pachytene chromosomes to demonstrate periodic arrangements of active and repressive histone marks, further underscoring nanoscale epigenetic compartmentalization^17^.

In live cells, Nozaki et al. applied photoactivated localization microscopy (PALM) to visualize chromatin domains that appeared as compact, dynamic structures ~160–220 nm in diameter. These domains moved coherently and persisted through mitosis, acting as stable building blocks of chromosome organization^22^. Notably, their structural organization was influenced by cohesin and nucleosome-nucleosome interactions.

Xu et al. used STORM imaging to classify chromatin domains based on histone modifications, revealing three distinct spatial patterns: segregated nanoclusters (histone acetylation), dispersed nanodomains (active methylation), and compact aggregates (repressive methylation)^23^. Active transcription was spatially correlated with less compact chromatin, and repressive marks were found in densely packed regions^5,23^. Rahman et al. expanded the scale and dimensionality of super-resolution nuclear profiling by implementing 3D multiplexed Exchange-PAINT and HIST microscopy, showing that proteins and chromatin marks organize into nanoscale domains with distinct spatial scaling behavior^24^.

### Electron tomography validates dense chromatin domains

Electron microscopy has independently validated these structures. ChromEMT, developed by Ou et al., confirmed that chromatin in situ exists not as regular 30-nm fibers but as disordered chains of 5–24 nm fibers forming dense, irregular domains^20^. These domains varied in chromatin volume concentration (12–52%) and appeared consistent with nanodomains observed by STORM. Similarly, ChromSTEM^25^, with the capability to quantify chromatin mass at ~2–4 nm resolution, suggested characteristic fractal scaling and demonstrated that these structures persist after cohesin depletion, underscoring their physical rather than topological identity. Miron et al. used 3D-SIM and FIB-SEM to reveal that chromatin forms 200 to 300 nm domains, referred to as chromatin domains (CDs), which are arranged in chains separated by RNA-rich interchromatin space^26^. These domains exhibited a nanoscale zonation with repressive marks enriched in the interior and active transcription machinery localized to the periphery, further supporting a model of compartmentalized genome function.

### Imaging genomic loci and epigenomic states in context

Multiplexed FISH approaches such as Oligopaint have enabled spatial mapping of hundreds to thousands of genomic loci with epigenetic and transcriptional context. Boettiger et al. showed distinct folding principles for active, inactive, and Polycomb-repressed domains using Oligopaint-STORM, revealing divergent size scaling exponents and internal mixing patterns^5^. Micro-C^27^, a high-resolution Hi-C variant, has further revealed sub-TAD features that resemble nanodomains in their spatial scale and variability, especially under conditions of cohesin depletion. These findings align with recent multiplexed point-localization studies demonstrating that transcriptional and epigenetic targets stratify below ~300 nm, with histone acetylation marks (e.g., H3K27ac) forming nanoscale clusters that partition from repressive marks and DNA upon transcriptional inhibition or epigenetic drug treatment, underscoring their functional autonomy from broader compartments^24^.

It is important to acknowledge that super-resolution and EM measurements of chromatin domains are sensitive to fixation, labeling, and reconstruction, and some fixation protocols may perturb native organization, motivating cryo-based approaches. Reported domain sizes, therefore, reflect both technical constraints and biological variation across cell types and states; accordingly, for this review, we emphasize convergent features over absolute size estimates. Live-cell imaging of chromatin nanodomains^22^ partially mitigates these concerns, though broader and systematic studies are still needed to establish their generality.

## Physical and epigenetic architecture

Nanoscale chromatin domains represent not only structural units of genome organization but also epigenetically defined regulatory modules. Having established their existence, the next step is to understand their physical and chemical composition. Unlike looping driven chromatin segregation, these nanoscopic domains emerge from local nucleosome-level organization coupled to specific epigenetic states, as revealed primarily through super-resolution imaging. In the sections that follow, we consider their defining features: physical size and packing, epigenetic and transcriptional stratification, independence from loop extrusion and tethering to the nuclear and nucleolar peripheries.

### Domain size, packing, and fractality

Chromatin domains have been observed across imaging modalities^20,28,29^ and across various cell lines, including mesenchymal stem cells^30^, fibroblasts^31^, mouse cells^28^, T cells^32^, B cells^33^ and various human and mouse cancer cell types^23^. A consistent feature of these domains is their characteristic size distribution, with radii typically ranging from 50 to 200 nm and averaging around 100 nm. The distribution is heavy-tailed, indicating the presence of larger domains up to a micron, likely corresponding to highly condensed chromatin regions such as centromeric or telomeric zones^30,34,35^. Additionally, their internal structure is not homogeneous; and to characterize it in accordance with previously proposed fractal globule framework of the chromatin^36^, studies have fitted the observed domains against empirical power-law scaling of the chromatin mass with its radius, characterized by a fractal dimension (${D}_{f}$) of ~2.4–2.7^25^. This scaling suggests a spatial organization more compact than a random coil (${D}_{f}=2$) but less dense than a globule (${D}_{f}=3$). Super-resolution 3D imaging and correlation-based analyses (e.g., pair cross-correlation functions) have reinforced this picture, showing evidence of a heterogenous packing behavior for DNA and associated histone marks across scales from ~50 nm to 1 μm^24^. These domains also display chromatin volume concentration gradients: denser cores tend to be less accessible and are enriched in repressive features, while lower-density peripheries are more accessible and transcriptionally engaged^29^. Together, these observations establish nanoscale chromatin domains as structurally heterogeneous, fractal-like units whose size, packing, and internal density gradients provide a physical scaffold for differential accessibility and epigenetic regulation.

### Epigenetic and transcriptional stratification

**Fig. 2:**
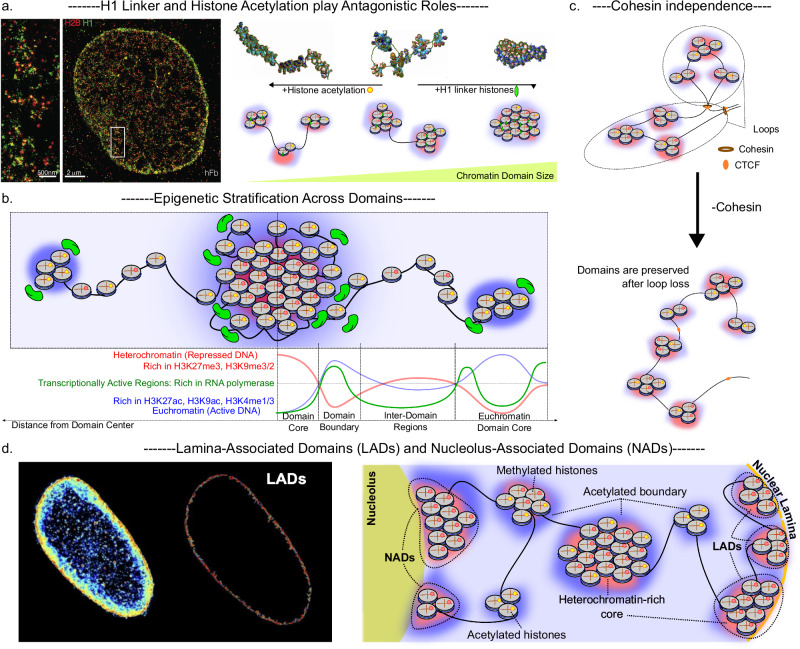
Physical and epigenetic organization of nanoscale chromatin domains. **a** Super-resolution imaging reveals H1 linker proteins are ubiquitous to chromatin nanodomains and act as antagonists to histone acetylation to control the size and packing density of domains^16,40^ **b** These domains exhibit radial stratification of epigenetic states, with transcriptionally active modifications (for example, H3K27ac, H3K4me3) and RNA polymerase II enriched at domain boundaries, while repressive marks (H3K27me3, H3K9me3) occupy the interior. **c** Absence of looping mechanisms does not affect domain size and distribution. **d** At the nuclear scale, chromatin nanodomains are tethered to architectural landmarks such as the nuclear lamina and nucleolus, forming lamina-associated domains (LADs) and nucleolus-associated domains (NADs). Together, these findings highlight that chromatin domains represent integrated physical-epigenetic modules, simultaneously defined by polymer packing principles and the spatial distribution of histone modifications. Note: The number of nucleosomes shown here is only representative, and they can vary from tens to a few hundred in a nanodomain.

Nanoscale domains exhibit epigenetic heterogeneity^16^ (Fig. 2b). Super-resolution imaging consistently shows a spatial separation of histone marks: active modifications such as H3K27ac and H3K4me3 localize toward the edges or peripheral shells of domains, often adjacent to interchromatin space. In contrast, repressive marks like H3K27me3 and H3K9me3 are enriched in the denser core regions and often associated with larger domains, particularly at the nuclear periphery^5,26^. The spatial positioning of these marks is functionally significant as well. Active transcription machinery (e.g., RNAPII, CDK9, p300) localizes preferentially near peripheral zones, which are rich in active chromatin markers, forming a local organization that supports rapid access and regulation^24,37^. Repressive zones within the domain interior often colocalize with structural proteins like HP1α or components of Polycomb complexes, suggesting a role in stabilizing silenced states through compaction and phase-like behavior^24^. Because enzymes that promote heterochromatin formation are generally smaller than those that facilitate euchromatin organization, domain maintenance may be further maintained by size-dependent diffusion: smaller heterochromatin enzymes can penetrate to the core, whereas larger euchromatin enzymes are restricted to the periphery, where they support transcription^26,38^. As we will discuss in the next section, this spatial partitioning, defined by boundary positioning, has significant biophysical underpinnings and is closely tied to transcriptional plasticity and cellular heterogeneity.

In addition to histone modifications, the distribution of linker histone H1 modulates domain architecture^16,39^ (Fig. 2a). Experimental depletion of H1 results in decreased packing efficiency and increased chromatin accessibility^16^. In silico studies further support this, showing that H1 occupancy is a major regulator of nucleosome domain compaction, working in concert with acetylation levels and nucleosome spacing^39,40^. Physical determinants of domain size and packing also include nucleosome spacing. The presence of nucleosome-free regions (NFRs) strongly influences domain formation. Increased NFR frequency correlates with smaller, more fragmented domains through reduced internucleosomal interactions^40^. Together, these findings establish that nanoscale domains are not passive structural aggregates but epigenetically and physically stratified modules, in which the interplay of histone marks, architectural proteins, enzyme accessibility, and nucleosome organization collectively governs compaction, accessibility, and transcriptional potential.

### Structural persistence without loop extrusion

A key distinction between nanoscale domains and the higher order structure is their independence from the loop extrusion machinery (Fig. 2c). ChromSTEM^25^ has shown that these domains display a characteristic scaling behavior and are preserved after RAD21 depletion^41^, indicating that their formation does not rely on loop extrusion. Similarly, Miron et al. found that the structure persists in the absence of cohesin, reinforcing the notion that these domains are physical, not just topologically extruded, units^26^. This is supported by single-cell 3D-SIM studies^42^ demonstrating that nanodomains within TADs remain intact in cells depleted of either CTCF or cohesin, despite a loss of TAD-level insulation. However, this independence does not imply that loop extrusion machinery plays no role in domain size selection. Recent studies suggest that cohesin- and RNAPII-driven supercoiling can modulate the size distribution of these domains, hinting at a nuanced interplay between transcriptional activity, torsional stress, and physical compaction^35,37^, an aspect we will cover from a biophysical perspective in the next section. Studies have established that chromatin loops can also arise through diffusion- or Brownian-capture mechanisms in which SMC complexes stabilize thermally generated loops rather than processively extruding them, both for cohesin in interphase chromatin and for condensin during mitotic compaction^43,44^. Our use of “independence from loop extrusion” therefore refers specifically to the persistence of these nanoscale domains when classical cohesin-dependent loop extrusion is compromised and is compatible with local SMC-mediated loop capture operating within or at the boundaries of the domains.

### Tethered chromatin domains at the lamina and nucleolus

Beyond the nuclear interior, compact chromatin domains are also observed at the nuclear periphery and around the nucleolus (Fig. 2d). Super-resolution imaging of mammalian cells reveals that lamina-associated domains (LADs), identified as discrete, heterochromatic structures, exhibit an epigenetic constitution that resembles domains in the nucleus interior^28,30,31,45^. These peripheral domains vary in thickness and chromatin density depending on their epigenetic state and the presence of interacting proteins, such as HDAC3 and LAP2β^46–48^. While nucleolus-associated domains (NADs) remain less well characterized at super-resolution, their enrichment in heterochromatin and positioning near the nucleolar periphery allude that they may follow similar principles^49–51^. These findings suggest that local interactions and epigenetic states, operating through the same core biophysical principles that govern chromatin domains, could contribute to the organization of compact chromatin structures along the nuclear periphery as well.

Despite extensive imaging-based observations, these domains are still often treated as distinct structural entities rather than elements of a unified framework. Their physical and epigenetic features are well described, yet how these layers integrate into a coherent model of chromatin organization remains unclear. Critical questions persist, including the causal links between packing density, histone mark localization, and the functional outcomes of these universal packing principles. In the next section, we explore emerging conceptual models that aim to reconcile these findings, offering an integrative view of chromatin architecture at the nanoscale and its role in enabling cells to regulate phenotypic trajectories. Box 1.

Box 1 How Single-Molecule Localization Microscopy (SMLM) works: a physics perspective **The diffraction barrier**: In a conventional light microscope, objects closer than ~200–300 nm blur into one spot because of the diffraction limit. This limit comes from the wave nature of visible light: when light passes through a finite lens aperture, it diffracts, producing an Airy disk pattern rather than a perfect point. The smallest resolvable separation is given approximately by Abbe’s formula, $d\approx \lambda /(2{NA})$, where $\lambda$ is the emitted wavelength of the fluorophore (~450–700 nm), and NA is the numerical aperture of the lens. In practice, achievable resolution is often worse than this theoretical limit due to aberrations, background, and sampling constraints. The intuitive notion of two nearby points merging into one corresponds more precisely to the Rayleigh criterion, which provides a closely related but distinct resolution estimate. An everyday analogy is two distant car headlights: if they are too close together, diffraction makes them blur into a single bright spot.

**Principle of SMLM**^10^: The insight behind SMLM (which encompasses STORM^15^, PALM^103^, DNA-PAINT^104^, and other similar techniques) is that it is not necessary to resolve all molecules at once; only their individual positions need to be determined. Instead of all fluorophores emitting simultaneously, only a sparse random subset is switched “on” at a time. Each active molecule appears as a blurred spot (a point-spread function), which can be fitted mathematically to determine its center with nanometer precision. This localization precision depends on photon statistics and improves with the number of detected photons $N$ (error scales as $1/\surd N$). *This is like listening to people in a noisy room: if everyone speaks at once you cannot distinguish voices, but if only one person speaks at a time you can pinpoint their location with great precision and the greater number of times they speak, the more certain you are about their position*. By repeating many cycles of stochastic activation and localization, a high-resolution image is reconstructed from the accumulated coordinates. Typical SMLM experiments achieve ~20 nm resolution, an order of magnitude beyond conventional light microscopy.

**Recognition and impact**: The transformative significance of super-resolution methods was acknowledged by the 2014 Nobel Prize in Chemistry, awarded to Eric Betzig, Stefan Hell, and William Moerner, and followed by the 2019 Breakthrough Prize in Life Sciences to Xiaowei Zhuang. Following the initial overturning of the classical-30 nm fiber view of chromatin^13,105^ STORM directly revealed that nucleosomes organize into heterogeneous “clutches” and nanometer-scale domains.

## Understanding the physics of domain regulation

The structural features of nanoscale chromatin domains, including their compact organization, radial stratification, and independence from loop extrusion, raise important questions about the molecular and physical mechanisms that govern their formation and stability. Physical modeling has been instrumental in unifying the multiple scales of chromatin organization and in developing a mechanistic understanding of their underlying principles^52–58^. Although imaging studies have characterized their morphology and compositional complexity, a mechanistic understanding of how these domains are formed and regulated and how they influence the transcriptional state of the cell remains an open challenge. Emerging theoretical and computational models, informed and validated by experimental data, suggest that these domains are not passive consequences of chromatin folding. Instead, they appear to arise from the interplay of epigenetic reaction-diffusion processes, transcriptional activity, and spatial anchoring to nuclear structures. Within this framework, chromatin domains are best viewed as steady-state attractors in a dynamic, energy-consuming landscape shaped by both biochemical and physical constraints. The underlying physical principles that have been used to understand domain size, positioning, and morphology distributions are recapitulated in Box 2.

Box 2 Physical principles behind nanoscale chromatin domains Three physical principles underlie chromatin nanodomain organization: diffusion-driven phase separation, non-equilibrium reaction–diffusion balance, and wetting at nuclear surfaces. Together, these mechanisms explain how domains form, stabilize at finite size, and position within the nucleus.

**Diffusion and phase separation:** Diffusion spreads molecules and erases concentration differences, with a characteristic timescale $t\sim {L}^{2}/D$, where ${L}$ is the distance traveled and $D$ the diffusion coefficient. In the nucleus, this implies that protein gradients over micron-length scales dissipate within seconds. However, if molecules preferentially bind to one another, attractive interactions can amplify local fluctuations instead of smoothing them, driving phase separation. Under equilibrium conditions, this leads to coarsening (Ostwald ripening): small clusters dissolve, and larger ones grow, progressively increasing droplet size. Attractive nucleosome–nucleosome and nucleosome-epigenetic factor interactions can therefore segregate heterochromatin and euchromatin. Yet equilibrium phase separation predicts indefinite growth; the observed 50–200 nm domain sizes indicate that coarsening must be actively arrested.

**Non-equilibrium activity and reaction-diffusion balance:** Cells break equilibrium by consuming energy. Active histone-modifying reactions can write and erase epigenetic marks. This creates a non-equilibrium steady state, analogous to a sink with continuous inflow and outflow: domain size appears stable because diffusion is opposed by active reactions, and hence opposing fluxes are balanced. Diffusion delivers heterochromatin into a domain at a rate scaling as $D/R$ (where $R$ is domain radius), whereas enzymatic turnover removes or remodels chromatin at a rate scaling as $\Gamma R$ (where $\varGamma$ is an effective reaction rate). Balancing these fluxes yields a steady-state radius ${R}_{{ss}}\sim \sqrt{D/\Gamma }$, fixing domains at a reproducible nanoscale size (~100 nm). Energy consumption thus halts the unlimited coarsening expected at equilibrium and stabilizes domains within a defined size range.

**Wetting and surface interactions:** Domain morphology and positioning are further governed by wetting. When a droplet contacts a surface, its shape reflects the balance between cohesion (internal attraction) and adhesion (interaction with the surface), described by Young’s law through the contact angle. Within the nucleus, the lamina and nucleolus (among other inclusions) act as adhesive substrates. Tethering interactions can therefore flatten, stabilize, or spatially anchor chromatin domains, producing lamina- or nucleolus-associated structures whose thickness reflects the balance between adhesion and reaction–diffusion dynamics.

### Domain emergence and maintenance through epigenetic reaction-diffusion competition

**Fig. 3:**
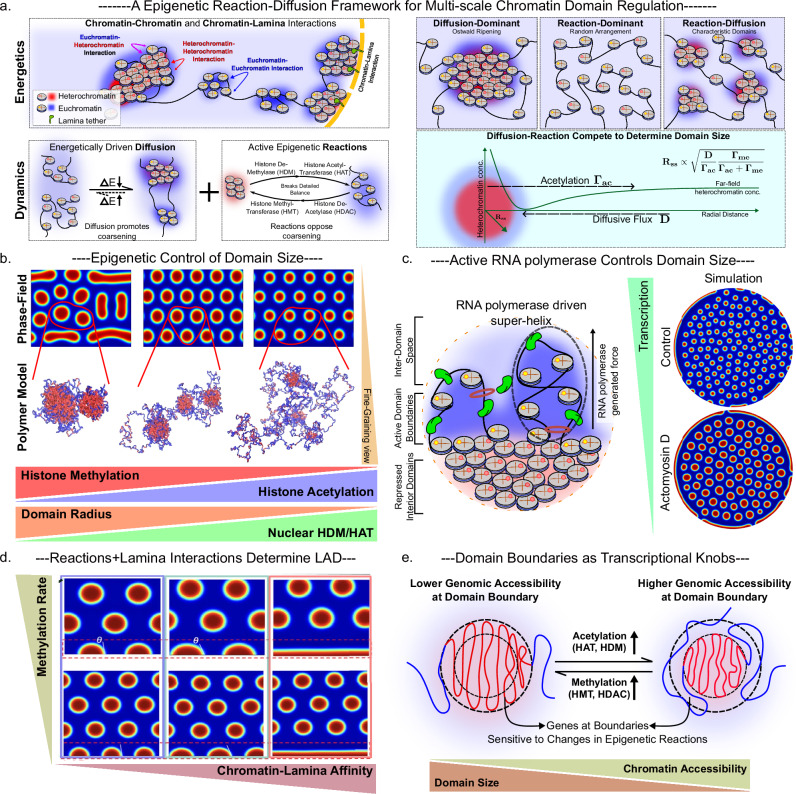
A unified biophysical framework for the formation, regulation, and functional consequences of chromatin nanodomains. Chromatin nanodomains arise from the interplay of diffusion, epigenetic reactions, polymer dynamics, transcriptional forces, and spatial tethering. **a** Chromatin-chromatin (internucleosome) and chromatin-lamina interactions create an energetic landscape favoring heterochromatin condensation and peripheral localization. The steady-state domain size is determined by the balance between diffusive influx and reaction-driven efflux at the domain boundary, leading to different regimes of domain size depending on relative reaction and diffusion strengths. **b** Theory-driven phase field and polymer simulations capture the scaling behavior of chromatin domains with changing epigenetic reaction rates. **c** RNA polymerase II imposes mechanical flux of chromatin out of domains via supercoiling and loop extrusion, locally driving chromatin decompaction at domain boundaries. **d** Chromatin-lamina affinity constrains domain positioning, leading to distinct LAD morphologies based on tethering strength (chromatin-lamina affinity) and methylation activity. The morphologies formed can thereby be characterized in terms of the LAD thickness and contact angles $\theta$. **e** The domain boundary serves as a site of switchable chromatin accessibility and transcriptional regulation, where genes can dynamically transition between repressed and active states in response to changes in histone writer/eraser driven active epigenetic reaction kinetics.

Polymer simulations^59^ and theoretical analysis^30^ have suggested that chromatin nanodomains form and persist through a constant tug-of-war between two fundamental dynamic processes that together define their characteristic size. First, passive diffusion drives nucleosomes carrying the same chemical tags, such as methyl groups on H3K9 or H3K27, toward one another, causing small clusters to merge into larger condensates. Such processes can be facilitated by linker proteins such as HP1 or due to electrostatic interactions^60^ (Fig. 3a). Left alone, this would lead to complete phase separation by Ostwald ripening. In living cells, however, histone epigenetic read-write enzymes (for example, methyltransferases like EZH2 and deacetylases such as HDACs) continually erase and install epigenetic marks, consuming metabolic cofactors (such as acetyl-CoA) in the process^61^. To unify this understanding, in the proposed models, these active processes are explained through two main processes: (a) Methylation, which refers specifically to repressive histone methylation pathways, primarily H3K9me3 and H3K27me3, and to associated processes that increase chromatin compaction or reduce transcriptional likelihood (including deacetylation). This terminology does not include activating methyl marks such as H3K4me3 or H3K36me3. DNA methylation can also contribute to compaction but operates through distinct mechanisms and timescales. (b) Acetylation, which refers to processes which decompact chromatin through deposition of marks such as H3K27ac and H3K9ac. Through acetylation, by converting heterochromatin back to euchromatin inside emerging droplets, these energy-consuming reactions generate a chemical flux that counterbalances the inward, energetically favourable diffusive flux of heterochromatin, arresting droplet growth and stabilizing domains at a characteristic nanometer scale (Fig. 3a). Because installing or removing an unfavorable mark, for example, placing a euchromatin mark within a heterochromatin-rich environment, requires energy, chromatin nanodomains should be understood as active, energy-driven structures rather than passive thermodynamic minima.

A biophysical scaling argument from these studies^34,35^ captures how these opposing fluxes set the steady-state radius ${R}_{{ss}}$ for nanodomains. As heterochromatic segments diffuse inward at a flow determined by an effective diffusion coefficient $D$, enzyme-driven heterochromatin mark removal (or euchromatin mark deposition) at rate ${\varGamma }_{{ac}}$ pushes chromatin back out. Detailed analytic derivations, confirmed by detailed phase-field^30,34,35^ and polymer simulations^59^ demonstrate that (Fig. 3b):

$${R}_{{ss}}\propto \sqrt{\frac{D}{{\varGamma }_{{ac}}}\frac{{\varGamma }_{{me}}}{{\varGamma }_{{me}}+{\varGamma }_{{ac}}},}$$

where ${\Gamma }_{{me}}$ is the methylation rate, which accounts for repressive heterochromatin mark deposition (or euchromatin mark removal). In simpler terms, higher diffusion or methylation activity supplies more heterochromatin to the growing droplet, enlarging it, whereas greater demethylation activity removes marks more rapidly, shrinking the domain. Through experimentally validated epigenetic remodeler concentrations within the nucleus, simulations achieve nanodomain sizes of approximately 100 nm, in striking agreement with distributions exhibited by super-resolution imaging across cell lines. These findings are further supported by epigenetic drug treatments, including Trichostatin A (TSA), a broad HDAC inhibitor, and GSK343, an EZH2 inhibitor, with consistent effects observed across human and mouse mesenchymal, epithelial, and cancer cell lines^30,31,59^.

The reaction-diffusion framework can be further extended to specify how changes to the underlying chromatin fiber architecture can effectively determine chromatin architecture. Portillo-Ledesma et al.^40^ simulate mesoscale chromatin “clutches” and show that local compaction is sensitive to fiber-level parameters: linker histone H1 density and nucleosome-free region (NFR) length. Their findings provide a structural basis for modulating the effective rates of methylation (${\varGamma }_{{me}}$) and acetylation (${\varGamma }_{{ac}}$) in reaction-diffusion models, for example, tighter clutches formed under high H1 correspond to higher effective methylation activity, while long NFRs reduce compaction and exhibit the opposing effect. These structural effects can be folded into the scaling law discussed above, showing how local chromatin organization tunes global domain size. Together, these findings establish chromatin nanodomains as steady-state products of an energy-consuming reaction–diffusion balance that is further tuned by polymer-level features of the chromatin fiber. By linking enzymatic activity, metabolite availability, and nucleosome-scale organization to reproducible nanoscale structures, this framework provides a mechanistic bridge between molecular biochemistry and nuclear architecture, offering a unified physical basis for understanding how cells control mesoscale genome compaction and accessibility.

### Transcription-coupled regulation of nanodomain size

Domain boundaries provide a natural spatial niche for transcription, serving as sites where RNA polymerase and associated machinery are preferentially localized. Here, we turn to the role of this transcriptional machinery itself, not just as a responder to chromatin state, but as an active regulator that shapes the transcriptional landscape of the cell. Beyond the balance of diffusion and epigenetic reactions, RNA polymerase II (RNAPII) adds a mechanical layer of control: as polymerases transcribe DNA, they generate negative supercoils that drive loop extrusion at the heterochromatin-euchromatin boundary, held at the hinge by cohesin (Fig. 3c). Mechanistically, this process acts like a tug on the droplet’s surface, pulling away repressive chromatin and transforming it to active chromatin. Kant et al.^35^ captured this in a phase-field model by adding an extrusion-driven flux (rate ${\Gamma }_{a}$) to the previously described reaction-diffusion system. The result is a modification of the reaction-diffusion steady-state radius:

$${R}_{{ss}}\propto \sqrt{\frac{D}{{\varGamma }_{{ac}}+{\varGamma }_{a}}\frac{{\varGamma }_{{me}}}{{\varGamma }_{{me}}+{\varGamma }_{{ac}}+{\varGamma }_{a}}.}$$

In practice, the extrusion-driven flux (${\varGamma }_{a}$) is a function of the cohesin residence time (${\varGamma }_{{coh}}$), and thereby its loading and unloading and the rate of transcription by RNAPII (${\varGamma }_{{tr}}$), which act in a dependent manner, thereby ${\varGamma }_{a}={\varGamma }_{{coh}}\times {\varGamma }_{{tr}}$. The authors validate this model through experimental data that inhibited WAPL (a cohesin unloader) and RNAPII and observed the corresponding domain shrinkage and growth. These results demonstrate that transcriptional forces act as a “tunable knob” for chromatin nanodomains across cell lines^35,37^. This positions transcription as an active participant in shaping chromatin architecture, rather than a downstream consequence of it. By coupling RNA polymerase activity to loop extrusion, cells gain a mechanism to dynamically modulate domains, specifically at their boundaries, and fine-tune gene accessibility in response to changing transcriptional demands.

### Spatial anchoring and formation of lamina and nucleolus associated domains

Peripheral chromatin domains form when heterochromatin is anchored to nuclear structures like the lamina or nucleolus through specific tethering proteins such as LAP2β, LBR, and Emerin^48^ (Fig. 3a). Dhankhar et al.^34^ extended the reaction–diffusion framework by incorporating chromatin–lamina adhesion, providing a physical model for the formation and shaping of LADs (Fig. 3d). In this model, LAD size emerges from the balance between chromatin diffusion, enzymatic modification rates, and the strength of tethering to the nuclear lamina. Stronger adhesion leads to broader, sheet-like LADs that spread along the nuclear periphery, whereas weaker interactions produce thinner, punctate structures (with the spreading defined through the contact angle $\theta$, explained in Box 2). These predictions are supported by super-resolution imaging, which reveals a bimodal distribution of laminar affinity, likely corresponding to low affinity near nuclear pores and high in regions enriched with anchoring proteins such as LBR and LAP2β. Importantly, the model draws from classical wetting physics: the morphology of LADs is governed by effective surface tensions between chromatin states and the nuclear lamina, much like how a droplet spreads on a solid surface. The contact angle $\theta$ in this analogy reflects the energetic favorability of peripheral chromatin association. Thus, LADs are not rigid compartments but dynamic structures whose size and shape adapt to biochemical and mechanical cues. Consistent with this view, live-cell super-resolution imaging shows that tethering not only affects the spreading behavior of LADs but also constrains their motion, linking spatial anchoring to the regulation of chromatin mobility and nuclear organization^22^.

By analogy, nucleolus-associated domains (NADs) form via tethers like nucleolin and NPM1 that bind repressive chromatin to the nucleolar surface^62^ (Fig. 3a). Although super-resolution data on NADs remain less analyzed, natural extensions of current theoretical models predict that adding a similar adhesion term for nucleolar tethers recapitulates the heterochromatin clusters observed around the nucleolus. Mechanistically, both LADs and NADs demonstrate how active epigenetic reactions, such as methylation and acetylation, generate nanodomains whose size, shape, and peripheral positioning are stabilized by specific protein–membrane interactions. In this way, non-equilibrium events are converted into stable, transcriptionally silent compartments. This principle is particularly significant given that nearly 40% of chromatin is associated with the nuclear periphery, underscoring its central role in large-scale gene silencing^63^.

### Domain boundaries as sites of epigenetic remodeling and transcriptional control

A key feature of reaction-diffusion models is that chromatin nanodomains develop non-uniform internal structure, with biochemical activity localized at their edges. As repressive marks like H3K9me3 accumulate through inward diffusion and nucleosomal attraction, a densely packed core forms that is energetically stable and largely inaccessible to enzymatic remodeling. Surrounding this core, however, is a depletion layer: a peripheral region where chromatin is less compact, physically accessible, and most acetylated (Figs. 2b and 3a). Simulations show that this layer naturally emerges from the coupling of polymer mechanics and reaction kinetics and serves as the primary site of epigenetic turnover. Because modifying the core would require breaking favorable heterochromatin-heterochromatin interactions, mark removal reactions, such as acetylation or demethylation, occur primarily at the boundary, where the energetic cost is lower, and enzymes can more easily access the chromatin fiber. This creates a localized reaction zone that counteracts the inward flux of heterochromatin, stabilizing domain size and composition. The model thus predicts^34,35,59^, and imaging confirms^26^, that euchromatic marks and transcriptional regulators are enriched at domain boundaries, where physical accessibility and epigenetic responsiveness converge.

This boundary-localized remodeling has important implications for transcriptional plasticity. Because epigenetic reaction-driven gene accessibility alterations are concentrated at domain edges, genes positioned near these boundaries are uniquely poised to respond to fluctuations in reaction rates. Simulations show that changes in the rates of methylation or acetylation—whether driven by shifts in enhanced expression of epigenetic remodelers, changes to the cell metabolic state, or through external chemo-mechanical signaling leading to shuttling of enzymes to the nucleus—can lead to rapid and localized changes in domain size, disproportionately affecting chromatin at the periphery. As the boundary moves outward or inward, genes near this interface can be brought into or pushed out of a repressive environment without requiring global chromatin reorganization. This creates a highly tunable mechanism for controlling gene accessibility: boundary-proximal genes can switch transcriptional states quickly, while those buried deep within domains remain insulated from transient fluctuations (Fig. 3e). Such spatial compartmentalization of responsiveness enables the cell to maintain stable gene silencing at the core while preserving reactive capacity at the edge, offering a physical basis for gene silencing through compaction and rapid epigenetic adaptation in response to developmental cues or environmental stress. In the next section, we will discuss how such localized regulation manifests in desirable ways in pluripotency and undesirable ways in cancer. Although both states exhibit high transcriptional plasticity, arising from smaller domain cores and increased effective surface area, pluripotent cells use this flexibility to pursue multiple developmental trajectories, whereas cancer cells exploit it to withstand stress and acquire drug tolerance.

Concludingly, nanoscale chromatin domains emerge from an active balance of epigenetic reactions, transcriptional forces, and nuclear tethering. Reaction–diffusion models provide a mechanistic framework for how domain growth is arrested, transcription modulates domain size, and anchoring fixes spatial position within the nucleus. In parallel, alternative approaches based on self-returning polymer dynamics^64–66^ have also recapitulated observed domain geometries and hypothesized fractal-like scaling behavior, but they omit essential factors such as non-equilibrium enzymatic activity and environmental responsiveness. These models offer a valuable geometric reference but overlook the dynamic, regulated nature of chromatin domains in living cells. A major outstanding question is whether the proposed fractal-like scaling of these domains^24,25^ is truly observed at multiple scales and to understand their causality. Resolving this will require multiscale, high-fidelity imaging combined with perturbative experiments and integrative modeling to bridge physical structure with biological function.

## Functionality and responsiveness of the domains through development, disease, and extracellular cues

The previous section outlined a working model in which active reactions, driven by epigenetic write/erase enzymes (such as HDACs, HMTs, HATs, and HDMs), define the global size distribution of the nanoscale chromatin domains. Here, we examine how this cascade affects the phenotypic trajectories of the cell. As established, through computational and theoretical modeling, rather than static bundles of DNA, chromatin domains can act as modular control units: the outer shell favors transcription, the inner core maintains repression, and the balance between the two shifts signals (reflected through the epigenetic reaction rates). Such shifts can occur when the cell encounters changes through processes such as extracellular mechanical stress, metabolic change, or developmental signals. The case studies that follow—spanning pluripotency, immune activation, extracellular cues, and cancer—illustrate how alterations in domain size distribution, particularly boundary-driven transcription control, translate into changes in gene expression and cell identity. They also highlight where the current framework succeeds and where new mechanistic are needed.

### Domain distribution response to extracellular mechanical and chemical cues

**Fig. 4:**
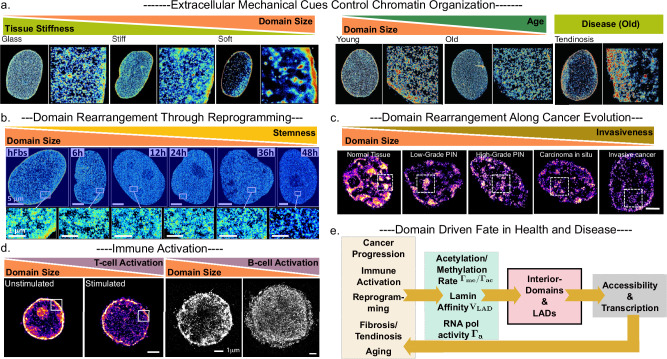
Chromatin domain remodeling as a unifying principle across biological contexts. Super-resolution imaging reveals that domain size and boundary organization change systematically in response to diverse physiological and pathological cues. **a** Human mesenchymal stem cells on stiff substrates show smaller, more dispersed domains, whereas soft matrices or hypoxia promote compaction and thickened lamina-associated domains (LADs)^30^. Aging also leads to changes in condensation where domain sizes decrease with age, while tendinosis produces a distinct nuclear phenotype with persistent peripheral LADs and coarse interior domains, highlighting the divergence between degeneration and aging^30^. **b** During heterokaryon-driven reprogramming of human fibroblasts, domain remodeling unfolds over days, with progressive chromatin decompaction and fragmentation of repressive cores restoring pluripotency^71^. **c** In cancer progression, domains gradually decompact and fragment, transitioning from normal tissue through premalignant lesions to invasive cancer, with early loss of compact cores and boundary integrity^24,74^. **d** Immune activation exploits domain plasticity, with both T- and B-cell stimulation triggering domain fragmentation, rim broadening, and expansion of transcriptionally active nuclear space^24,32,33^. **e** Schematic: Diverse outcomes can be unified under a reaction-diffusion feedback framework, where shifts in methylation/acetylation balance (${\varGamma }_{{me}}/{\varGamma }_{{ac}}$), lamina affinity (${V}_{{LAD}}$), and RNA polymerase activity (${\varGamma }_{a}$) regulate domain size and LAD organization, thereby controlling gene accessibility and transcriptional output.

Microenvironmental cues such as substrate stiffness, oxygen tension, and inflammatory signals modulate chromatin domain architecture by altering epigenetic writer/eraser concentrations in the nucleus^30^. Super-resolution imaging reveals that softer environments increase domain sizes in hMSCs, possibly through H3K27me3-driven epigenetic regulation, also leading to peripheral condensation, i.e., increase of lamina-associated domains (LADs) (Fig. 4a). Hypoxic stress also leads to an increase in the domain size^30,67^. Quantitatively, soft 3 kPa matrices or 1 % O₂ increase mean LAD thickness from 70 nm to 150 nm and enlarge interior clusters by ~40 %. These changes are hypothesized to arise through cytoplasm-to-nucleus shuttling of epigenetic remodelers like HDACs and EZH2 (leading to an effective increase in methylation rates, ${\varGamma }_{{me}}$), a mechanism that may serve as a general conduit linking external inputs to transcriptional control^68,69^. Such shifts in modification rates reshape domain size distributions and spatially reorganize open and closed chromatin, thereby altering transcriptional potential and cell identity.

Tendinosis imposes a distinct nuclear phenotype that differs from normal aging (Fig. 4a). In degenerative tenocytes, chromatin relocalizes to the periphery and condenses into thickened LADs, while interior domains remain coarse even when stiffening cues are applied^30^. Aged cells, in contrast, retain partial decondensation capacity, indicating that degeneration and aging produce divergent architectural outcomes. Within the reaction-diffusion framework, tendinosis reflects trapping in a high-${\varGamma }_{{me}}$, high lamina-affinity state, whereas healthy and aged cells can still shift domain distributions. This loss of mechanoresponsive plasticity distinguishes chronic degeneration from gradual aging and positions domain remodeling as a central mechanism in tendon disease.

### Evolution of domains through cellular reprogramming

During developmental transitions such as differentiation and reprogramming, chromatin domains undergo pronounced reorganization that both reflects and enables shifts in transcriptional identity. Embryonic stem cells harbor small, diffuse domains with low compaction, enriched in active histone marks^25,28^. As differentiation proceeds, domains coalesce into larger, denser structures driven by rising linker histone H1 level, promoting heterochromatin formation and restricting transcriptional plasticity. Within the theoretical framework, this increases the effective methylation to acetylation ratio (${\varGamma }_{{me}}/{\varGamma }_{{ac}}$), contracts the accessible chromatin, and yields larger, denser domains that restrict transcriptional plasticity in a boundary-driven manner. Reprogramming to induce pluripotency reverses these changes: repressive cores fragment, active rims expand, and chromatin becomes more accessible at key loci such as *Nanog*^16,70^ (Fig. 4b). Martínez-Sarmiento et al. showed that the Nanog-associated domain decompacts before Nanog transcription increases, underscoring the regulatory importance of local chromatin domain architecture. Consistent with this principle, super-resolution imaging of heterokaryons reveals a gene-specific sequence of events: an increase in global acetylation leading to changes at the Nanog loci, which first undergoes local domain opening and later displays enhanced expression^71^. These findings confirm that chromatin domain remodeling acts at both gene-specific and global scales, regulating pluripotency-associated loci as well as broader transcriptional programs during reprogramming.

### Role of domains in cell transition to cancer

Nanoscale chromatin remodeling is emerging as a universal hallmark of cancer, characterized by progressive decompaction and redistribution of higher-order chromatin structures (Fig. 4c). Super-resolution imaging in both human and mouse tissues reveals that during early carcinogenesis, even in phenotypically normal cells, domains gradually lose their compact cores, leading to global chromatin decompaction and segregated boundaries, transitioning toward fragmented nanoclusters with reduced H3K9me3 and increased euchromatic signatures^72,73^. In the reaction-diffusion framework, this shift implies a sustained rise in the euchromatin-favoring reaction rate relative to the condensation rate $({\varGamma }_{{ac}}/{\varGamma }_{{me}})$, driving domains toward the high-plasticity region of parameter space. This evolution in domain topology is conserved across tumor types, including colorectal, prostate, and pancreatic cancers, and proceeds in a stepwise fashion from precancerous lesions to invasive tumors. In ovarian cancer, cancer stem cells (CSCs) exhibit increased numbers of poised and transcriptionally active domains, smaller clutch size, which correlates with greater transcriptional plasticity and chemotherapy resistance^74,75^. Notably, chromatin domain decompaction often precedes overt changes in gene expression or nuclear morphology, suggesting that nanoscale architecture serves as an early enabler of transcriptional deregulation and genomic instability^72^. Together, these findings position chromatin domain disruption as a globally conserved and functionally significant process that underlies the plasticity and progression of malignant states.

### T and B-cell activation

Immune stimulation exploits chromatin-domain plasticity to deliver rapid transcriptional alterations. In B cells, antigen engagement elevates Myc and acetyl-CoA synthesis, raising the histone-acetylation rate $({\varGamma }_{{ac}})$. Super-resolution studies show that peripheral heterochromatin nanodomains fragment, nucleoplasmic active space expands, and enhancer–promoter loops proliferate, supporting the surge in immunoglobulin transcripts^33^ (Fig. 4d). T-cell receptor signaling produces a similar response: chromatin decondenses genome-wide, median nanodomain diameter falls by roughly 40%, H3K4me3-rich rims, corresponding acetylated domain boundaries, broaden, and large RNA-polymerase assemblies accumulate, coincident with cytokine induction^32^ (Fig. 4d). Within the biophysical framework of “Understanding the physics of domain regulation,” both lineages undergo a transient shift toward the high-${\varGamma }_{{ac}}$, low-compaction corner of phase space, where smaller domains allow increased accessibility and maximize transcriptional plasticity. These examples underscore that regulated changes in domain size, compaction, and position form a conserved mechanism for state-specific gene activation in the immune system.

Together, these findings point to a unifying cascade that connects cell state with chromatin nano-architecture (Fig. 4e). Mechanical, metabolic, and developmental cues alter epigenetic reaction rates, lamin affinity, and transcriptional activity, which in turn reshape nanodomain distributions and the balance between active rims and repressive cores. When cells require plasticity, as in pluripotency, immune activation, or cancer, domains become smaller, more open, and less compacted, enabling broad transcriptional responsiveness. In contrast, differentiated or some degenerative states, like tendinosis, are marked by enlarged domains, thicker LADs, and stronger compaction, restricting accessibility. This simple framework suggests that chromatin domains act as universal regulatory units that translate molecular kinetics and physical compaction into transcriptional potential and determine whether cells remain plastic or committed.

## Conclusion and perspectives

The body of evidence reviewed here positions nanoscale chromatin domains—clutches, nanodomains, or packing domains—as fundamental building blocks of nuclear organization. Super-resolution and correlative electron microscopy revealed compact, heterogeneous chromatin clusters well below the diffraction limit, conserved across mammalian cell types and species. Detailed characterization has since established their defining properties: radially stratified organization of histone marks, transcriptionally active rims enriched with RNA polymerase II and cofactors, repressive cores stabilized by linker histones and silencing complexes, and structural persistence even in the absence of cohesin. These units are distinct from TADs and loops, capturing aspects of genome folding that escape ensemble-averaged contact maps and diffraction-limited imaging. In parallel, studies in yeast and other systems show that cohesin- and condensin-mediated clustering of distal loci can assemble higher-order gene interaction networks and clusters^76,77^, adding a complementary connectivity layer which might also be present in the nanoscale architecture discussed here.

At the coarser scale, live-cell imaging^78^ and single-cell Hi-C^79^ show that cohesin/CTCF-associated loops are dynamic and probabilistic: loop anchors fluctuate, and the looped configuration is sampled intermittently, so sharp loops and TADs in ensemble Hi-C arise from time- and population-averaged contact biases. Chromatin nanodomains appear analogously “statistically stable”: chromatin remains partitioned into compact clusters with a characteristic size distribution throughout interphase, while individual loci stochastically move in and out of these clusters, and domains are transiently replaced by coarser mitotic compaction during division. Thus, nanodomains are stable as mesoscale features of the ensemble, even though their precise microscopic realization fluctuates within each cell and over time.

**Fig. 5:**
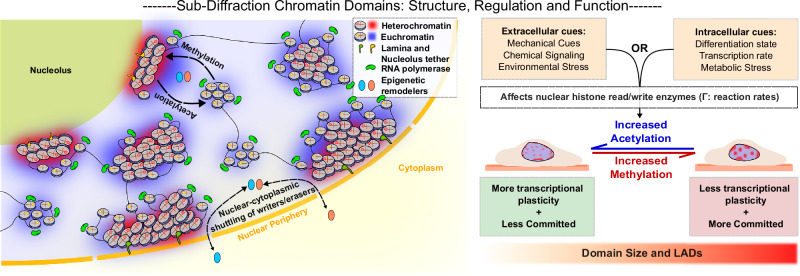
Chromatin nanodomains as integrators of nuclear organization, regulation, and cell fate. Chromatin is organized into nanoscale domains composed of transcriptionally active rims and repressive cores, stabilized through the interplay of epigenetic read/write enzymes, transcriptional forces, and nuclear tethering. These domains act as steady-state attractors in an active chromatin landscape, where diffusion-driven clustering is balanced by enzymatic turnover of histone marks. Intracellular factors (such as transcription rate, differentiation state, and metabolic stress) and extracellular inputs (including mechanical cues, signaling, and environmental stress) regulate nuclear availability of epigenetic remodelers, thereby shifting reaction rates that tune domain size and lamina association. The balance between acetylation and methylation determines whether domains favor transcriptional plasticity and responsiveness, or compaction and commitment. Through this dynamic regulation, nanoscale domains serve as modular control units that couple environmental and intracellular cues to transcriptional programs, epigenetic memory, and ultimately, cell identity. Nanodomains are drawn as compaction of a contiguous nucleosome array for schematic clarity.

Beyond descriptive characterization, a central insight is that the diverse processes shaping nanodomains are fundamentally unified within an active chromatin landscape (Fig. 5). Enzymatic read/write reactions, diffusion-driven clustering, transcription-induced supercoiling, and tethering to nuclear lamina (and nucleoli) come together to converge on the fundamental principle: chromatin domains are stabilized as steady-state attractors of out-of-equilibrium tugs-of-war. Reaction-diffusion models articulate how antagonistic fluxes of mark deposition and erasure arrest coarsening at nanoscale radii. Coupled with multiscale phase-field and polymer simulations, these frameworks reproduce the experimentally observed size distributions, radial stratification, and predict domain remodeling in response to epigenetic challenges, transcriptional disruption, or mechanical perturbation. Biophysical theory thus provides a scaffold integrating imaging, functional assays, and perturbation into a coherent model of genome regulation.

Across development, immune activation, mechanical stress, tissue degeneration, aging, and cancer, a consistent principle emerges: chromatin domains and their boundaries act as the nanoscale switches that set cellular trajectories. Core-rim stratification ensures that transcriptional activity is concentrated at boundaries, and modest shifts in epigenetic write/erase balance are sufficient to change rim positioning and alter epigenetic priming and gene accessibility. Increased methylation and higher lamina tethering promotes compaction, locking cells into repressive or committed states as seen in tendinosis or differentiation; increased acetylation expands rims and fragments cores, enhancing plasticity as in pluripotency, immune activation, or carcinogenesis (Fig. 5). In this sense, the fundamental biochemical processes of mark deposition and removal are amplified by the physics of domain organization into global control over plasticity and commitment. Boundaries, therefore, serve as the key integration points where non-equilibrium reaction-diffusion kinetics are converted into large-scale transcriptional malleability and phenotypic outcomes, making nanoscale domains the natural regulatory units through which cells navigate between stability and flexibility in phenotype.

Despite major advances, the key challenge is no longer establishing the existence of chromatin nanodomains but defining their generality, underlying mechanism, and functional logic. It remains unclear whether these structures represent an evolutionarily conserved organizing principle or a context-dependent feature of mammalian nuclei. Even their structural basis is unsettled: the proposed fractal-like scaling behavior^24,64^ lacks a clear mechanism, and the coexistence of spherical, elongated, and lamellar morphologies raises the possibility that domain geometry itself encodes regulatory information. Equally unresolved is the dynamic dimension. Nanodomains appear statistically stable, yet their nucleation, dissolution, and coupling to transcriptional bursting unfold over timescales that are only beginning to be quantified, limiting our ability to connect mesoscale architecture to regulatory kinetics.

A deeper tension lies in integration and causality. Imaging has richly described nanoscale structure, but its reconciliation with sequencing-based measurements remains incomplete, obscuring how physical domains map onto genome-wide enhancer-promoter interactions^80^ and transcriptional programs. As higher-resolution and deeper sequencing approaches begin to resolve increasingly fine-grained contact patterns, smaller subcompartments are emerging that approach the scale of imaging-defined nanodomains, raising the possibility that domains and compartments reflect different readouts of a shared underlying mesoscale organization rather than distinct entities^81^. Expanding the palette of perturbations to establish causality is critical. Although histone-modifying drugs are widely used, the effects of osmotic stress^82^, compressive load^68^, shear^83^, and tensile forces^84^ on the nucleus remain poorly characterized, despite their intrinsic roles in development and metastasis progression^85,86^. Likewise, DNA perturbations, such as DNA damage^77,87,88^ and topoisomerase activity^89,90^ at the nanoscale are understudied^91^. Finally, although suggestive evidence points to central roles in epigenetic memory^92^ and cellular heterogeneity^93^, the logic for whether and how nanoscale organization encodes history and produces divergent cell fates is elusive.

Theoretical frameworks will be essential in bridging these gaps. Mechanical models that link chromatin stiffness, nuclear viscoelasticity, and large-scale deformations are poised to clarify how physical stresses reshape domain architecture^94^. More broadly, models must integrate molecular specificity with mesoscale organization, resolving the coupled timescales of transcriptional dynamics and epigenetic modification. Moving beyond coarse-grained abstractions toward finer-grained and atomistic descriptions will be essential to capture the biochemical detail underlying histone modification, nucleosome interactions, and polymer folding^95^ Given the emerging links between domain remodeling and cancer, stem-cell plasticity, and degenerative disease, mechanistically grounded models carry direct translational relevance^96^ Precision in theory will be indispensable if nanoscale chromatin organization is to become therapeutically actionable.

Progress will depend equally on experimental convergence. High-resolution imaging integrated with spatially resolved transcriptomics will allow direct mapping of how domain remodeling propagates to gene expression in single cells. Advances in in situ structural approaches, including cryo-electron tomography^97,98^, promise to resolve domain architecture at molecular resolution. Systematic chemo-mechanical and synthetic perturbations, including CRISPR-based control of epigenetic marks^99^ will test causality rather than correlation. At the same time, interpretable machine learning frameworks^100–102^ offer a means to extract reproducible nanoscale patterns and distinguish mechanistic drivers from secondary effects. Together, these developments shift the field from descriptive cataloguing toward predictive and experimentally falsifiable models of chromatin organization.

A central goal is to establish a unified definition of *cell state*: one that merges nanoscale chromatin architecture with transcriptional and epigenomic profiles into a coherent, measurable entity. Achieving this genomic–physical picture at single-cell resolution will enable predictive models of cellular evolution, with implications for dormancy, drug resistance, neurodegeneration, and aging. Nanoscale chromatin domains lie at the core of this effort: as modular units through which the genome encodes responsiveness, memory, and fate, they represent the natural scale at which cellular identity is both stored and reshaped. By uniting molecular biology, high-resolution imaging, and single-cell sequencing with physically grounded theory and artificial intelligence, the field is poised not only to understand how domains structure the genome across space and time but also to harness this architecture to predict, and ultimately reprogram, cellular trajectories.

## References

[1] Jerkovic, I. & Cavalli, G. Understanding 3D genome organization by multidisciplinary methods. *Nat. Rev. Mol. Cell Biol.* **22**, 511–528 (2021). 33953379 10.1038/s41580-021-00362-w

[2] Li, H. et al. Chromosome compartmentalization: causes, changes, consequences, and conundrums. *Trends Cell Biol*. **34**, 707–727 (2024). 10.1016/j.tcb.2024.01.009 PMC11339242 38395734

[3] Bannister, A. J. & Kouzarides, T. Regulation of chromatin by histone modifications. *Cell Res.* **21**, 381–395 (2011). 21321607 10.1038/cr.2011.22 PMC3193420

[4] Wang, S. et al. Spatial organization of chromatin domains and compartments in single chromosomes. *Science* **353**, 598–602 (2016). 27445307 10.1126/science.aaf8084 PMC4991974

[5] Boettiger, A. N. et al. Super-resolution imaging reveals distinct chromatin folding for different epigenetic states. *Nature* **529**, 418–422 (2016). 26760202 10.1038/nature16496 PMC4905822

[6] McCord, R. P., Kaplan, N. & Giorgetti, L. Chromosome conformation capture and beyond: toward an integrative view of chromosome structure and function. *Mol. Cell* **77**, 688–708 (2020). 32001106 10.1016/j.molcel.2019.12.021 PMC7134573

[7] Burgess, D. J. Chromosome structure at micro-scale. *Nat. Rev. Genet.* **21**, 337 (2020). 32346116 10.1038/s41576-020-0243-y

[8] Fudenberg, G. & Mirny, L. A. Higher-order chromatin structure: bridging physics and biology. *Curr. Opin. Genet. Dev.* **22**, 115–124 (2012). 22360992 10.1016/j.gde.2012.01.006 PMC3697851

[9] Banigan, E. J. & Mirny, L. A. Loop extrusion: theory meets single-molecule experiments. *Curr. Opin. Cell Biol.* **64**, 124–138 (2020). 32534241 10.1016/j.ceb.2020.04.011

[10] Lelek, M. et al. Single-molecule localization microscopy. *Nat. Rev. Methods Primers* **1**, 39 (2021). 10.1038/s43586-021-00038-x PMC9160414 35663461

[11] Heintzmann, R. & Huser, T. Super-resolution structured illumination microscopy. *Chem. Rev.* **117**, 13890–13908 (2017). 29125755 10.1021/acs.chemrev.7b00218

[12] Joti, Y. et al. Chromosomes without a 30-nm chromatin fiber. *Nucleus* **3**, 404–410 (2012). 22825571 10.4161/nucl.21222 PMC3474659

[13] Vicidomini, G., Bianchini, P. & Diaspro, A. STED super-resolved microscopy. *Nat. Methods* **15**, 173–182 (2018). 29377014 10.1038/nmeth.4593

[14] Maeshima, K., Hihara, S. & Eltsov, M. Chromatin structure: does the 30-nm fibre exist in vivo? *Curr. Opin. Cell Biol.* **22**, 291–297 (2010). 20346642 10.1016/j.ceb.2010.03.001

[15] Rust, M. J., Bates, M. & Zhuang, X. Sub-diffraction-limit imaging by stochastic optical reconstruction microscopy (STORM). *Nat. Methods* **3**, 793–795 (2006). 16896339 10.1038/nmeth929 PMC2700296

[16] Ricci, M. A. et al. Chromatin fibers are formed by heterogeneous groups of nucleosomes in vivo. *Cell* **160**, 1145–1158 (2015). 25768910 10.1016/j.cell.2015.01.054

[17] Prakash, K. et al. Superresolution imaging reveals structurally distinct periodic patterns of chromatin along pachytene chromosomes. *Proc. Natl. Acad. Sci. USA* **112**, 14635–14640 (2015). 26561583 10.1073/pnas.1516928112 PMC4664314

[18] Sati, S. & Cavalli, G. Chromosome conformation capture technologies and their impact in understanding genome function. *Chromosoma* **126**, 33–44 (2017). 27130552 10.1007/s00412-016-0593-6

[19] Maeshima, K., Ide, S. & Babokhov, M. Dynamic chromatin organization without the 30-nm fiber. *Curr. Opin. Cell Biol.* **58**, 95–104 (2019). 30908980 10.1016/j.ceb.2019.02.003

[20] Ou, H.D. et al. ChromEMT: visualizing 3D chromatin structure and compaction in interphase and mitotic cells. *Science* **357**, eaag0025 (2017). 10.1126/science.aag0025 PMC5646685 28751582

[21] Lakadamyali, M. & Cosma, M. P. Visualizing the genome in high resolution challenges our textbook understanding. *Nat. Methods* **17**, 371–379 (2020). 32123395 10.1038/s41592-020-0758-3

[22] Nozaki, T. et al. Dynamic organization of chromatin domains revealed by super-resolution live-cell imaging. *Mol. Cell* **67**, 282–293 e7 (2017). 28712725 10.1016/j.molcel.2017.06.018

[23] Xu, J. et al. Super-resolution imaging of higher-order chromatin structures at different epigenomic states in single mammalian cells. *Cell Rep.* **24**, 873–882 (2018). 30044984 10.1016/j.celrep.2018.06.085 PMC6154382

[24] Rahman, F. et al. Mapping the nuclear landscape with multiplexed super-resolution fluorescence microscopy. *Nat. Commun.* **16**, 6042 (2025). 40593784 10.1038/s41467-025-61358-0 PMC12215941

[25] Virk, R. K. A. et al. Disordered chromatin packing regulates phenotypic plasticity. *Sci. Adv.* **6**, eaax6232 (2020). 31934628 10.1126/sciadv.aax6232 PMC6949045

[26] Miron, E. et al. Chromatin arranges in chains of mesoscale domains with nanoscale functional topography independent of cohesin. *Sci. Adv.* **6**, eaba8811 (2020). 10.1126/sciadv.aba8811 PMC7531892 32967822

[27] Krietenstein, N. et al. Ultrastructural details of mammalian chromosome architecture. *Mol. Cell* **78**, 554–565 e7 (2020). 32213324 10.1016/j.molcel.2020.03.003 PMC7222625

[28] Ricci, M. A., Cosma, M. P. & Lakadamyali, M. Super resolution imaging of chromatin in pluripotency, differentiation, and reprogramming. *Curr. Opin. Genet Dev.* **46**, 186–193 (2017). 28843811 10.1016/j.gde.2017.07.010

[29] Li, Y. et al. Analysis of three-dimensional chromatin packing domains by chromatin scanning transmission electron microscopy (ChromSTEM). *Sci. Rep.* **12**, 12198 (2022). 35842472 10.1038/s41598-022-16028-2 PMC9288481

[30] Heo, S. J. et al. Aberrant chromatin reorganization in cells from diseased fibrous connective tissue in response to altered chemomechanical cues. *Nat. Biomed. Eng.* **7**, 177–191 (2023). 35996026 10.1038/s41551-022-00910-5 PMC10053755

[31] Otterstrom, J. et al. Super-resolution microscopy reveals how histone tail acetylation affects DNA compaction within nucleosomes in vivo. *Nucleic Acids Res.* **47**, 8470–8484 (2019). 31287868 10.1093/nar/gkz593 PMC6895258

[32] Xu, J. et al. Super-resolution imaging of T lymphocyte activation reveals chromatin decondensation and disrupted nuclear envelope. *Commun. Biol.* **7**, 717 (2024). 38858440 10.1038/s42003-024-06393-1 PMC11164909

[33] Kieffer-Kwon, K. R. et al. Myc regulates chromatin decompaction and nuclear architecture during B cell activation. *Mol. Cell* **67**, 566–578 e10 (2017). 28803781 10.1016/j.molcel.2017.07.013 PMC5854204

[34] Dhankhar, M. et al. Revealing the biophysics of lamina-associated domain formation by integrating theoretical modeling and high-resolution imaging. *Nat. Commun.* **16**, 7909 (2025). 40854894 10.1038/s41467-025-63244-1 PMC12378204

[35] Kant, A. et al. Active transcription and epigenetic reactions synergistically regulate meso-scale genomic organization. *Nat. Commun.* **15**, 4338 (2024). 38773126 10.1038/s41467-024-48698-z PMC11109243

[36] Mirny, L. A. The fractal globule as a model of chromatin architecture in the cell. *Chromosome Res.* **19**, 37–51 (2011). 21274616 10.1007/s10577-010-9177-0 PMC3040307

[37] Neguembor, M. V. et al. Transcription-mediated supercoiling regulates genome folding and loop formation. *Mol. Cell* **81**, 3065–3081 e12 (2021). 34297911 10.1016/j.molcel.2021.06.009 PMC9482096

[38] Maeshima, K. et al. The physical size of transcription factors is key to transcriptional regulation in chromatin domains. *J. Phys. Condens. Matter.* **27**, 064116 (2015). 25563431 10.1088/0953-8984/27/6/064116

[39] Gomez-Garcia, P. A. et al. Mesoscale modeling and single-nucleosome tracking reveal remodeling of clutch folding and dynamics in stem cell differentiation. *Cell Rep.* **34**, 108614 (2021). 33440158 10.1016/j.celrep.2020.108614 PMC7842188

[40] Portillo-Ledesma, S. et al. Nucleosome clutches are regulated by chromatin internal parameters. *J. Mol. Biol.* **433**, 166701 (2021). 33181171 10.1016/j.jmb.2020.11.001 PMC7988292

[41] Li, W. S. et al. Mature chromatin packing domains persist after RAD21 depletion in 3D. *Sci*. *Adv*. **11**, eadp0855 (2025). 10.1126/sciadv.adp0855 PMC11759041 39854464

[42] Szabo, Q. et al. Regulation of single-cell genome organization into TADs and chromatin nanodomains. *Nat. Genet.* **52**, 1151–1157 (2020). 33077913 10.1038/s41588-020-00716-8 PMC7610512

[43] Uhlmann, F. A unified model for cohesin function in sister chromatid cohesion and chromatin loop formation. *Mol. Cell* **85**, 1058–1071 (2025). 40118039 10.1016/j.molcel.2025.02.005

[44] Kolbin, D. et al. Polymer modeling reveals interplay between physical properties of chromosomal DNA and the size and distribution of condensin-based chromatin loops. *Genes* **14**, 2193 (2023). 10.3390/genes14122193 PMC10742461 38137015

[45] Falk, M. et al. Heterochromatin drives compartmentalization of inverted and conventional nuclei. *Nature* **570**, 395–399 (2019). 31168090 10.1038/s41586-019-1275-3 PMC7206897

[46] Somech, R. et al. The nuclear-envelope protein and transcriptional repressor LAP2beta interacts with HDAC3 at the nuclear periphery and induces histone H4 deacetylation. *J. Cell Sci.* **118**, 4017–4025 (2005). 16129885 10.1242/jcs.02521

[47] Harr, J. C., Gonzalez-Sandoval, A. & Gasser, S. M. Histones and histone modifications in perinuclear chromatin anchoring: from yeast to man. *EMBO Rep.* **17**, 139–155 (2016). 26792937 10.15252/embr.201541809 PMC4783997

[48] Manzo, S. G., Dauban, L. & van Steensel, B. Lamina-associated domains: tethers and looseners. *Curr. Opin. Cell Biol.* **74**, 80–87 (2022). 35189475 10.1016/j.ceb.2022.01.004

[49] Padeken, J. & Heun, P. Nucleolus and nuclear periphery: velcro for heterochromatin. *Curr. Opin. Cell Biol.* **28**, 54–60 (2014). 24690547 10.1016/j.ceb.2014.03.001

[50] Bersaglieri, C. & Santoro, R. Genome organization in and around the nucleolus. *Cells* **8**, 579 (2019). 10.3390/cells8060579 PMC6628108 31212844

[51] Peng, T. et al. Mapping nucleolus-associated chromatin interactions using nucleolus Hi-C reveals pattern of heterochromatin interactions. *Nat. Commun.* **14**, 350 (2023). 36681699 10.1038/s41467-023-36021-1 PMC9867699

[52] Jost, D. et al. Modeling epigenome folding: formation and dynamics of topologically associated chromatin domains. *Nucleic Acids Res.* **42**, 9553–9561 (2014). 25092923 10.1093/nar/gku698 PMC4150797

[53] Abdulla, A. Z., Vaillant, C. & Jost, D. Painters in chromatin: a unified quantitative framework to systematically characterize epigenome regulation and memory. *Nucleic Acids Res.* **50**, 9083–9104 (2022). 36018799 10.1093/nar/gkac702 PMC9458448

[54] Fudenberg, G. et al. Formation of chromosomal domains by loop extrusion. *Cell Rep.* **15**, 2038–2049 (2016). 27210764 10.1016/j.celrep.2016.04.085 PMC4889513

[55] Owen, J. A., Osmanovic, D. & Mirny, L. Design principles of 3D epigenetic memory systems. *Science* **382**, eadg3053 (2023). 37972190 10.1126/science.adg3053 PMC11075759

[56] Shi, G. et al. Interphase human chromosome exhibits out of equilibrium glassy dynamics. *Nat. Commun.* **9**, 3161 (2018). 30089831 10.1038/s41467-018-05606-6 PMC6082855

[57] Di Pierro, M. et al. Transferable model for chromosome architecture. *Proc. Natl. Acad. Sci. USA* **113**, 12168–12173 (2016). 27688758 10.1073/pnas.1613607113 PMC5087044

[58] Zhang, B. & Wolynes, P. G. Topology, structures, and energy landscapes of human chromosomes. *Proc. Natl. Acad. Sci. USA* **112**, 6062–6067 (2015). 25918364 10.1073/pnas.1506257112 PMC4434716

[59] Vinayak, V. et al. Polymer model integrates imaging and sequencing to reveal how nanoscale heterochromatin domains influence gene expression. *Nat. Commun.* **16**, 3816 (2025). 40268925 10.1038/s41467-025-59001-z PMC12019571

[60] Erdel, F. & Rippe, K. Formation of chromatin subcompartments by phase separation. *Biophys. J.* **114**, 2262–2270 (2018). 29628210 10.1016/j.bpj.2018.03.011 PMC6129460

[61] Su, X., Wellen, K. E. & Rabinowitz, J. D. Metabolic control of methylation and acetylation. *Curr. Opin. Chem. Biol.* **30**, 52–60 (2016). 26629854 10.1016/j.cbpa.2015.10.030 PMC4731252

[62] Bizhanova, A. & Kaufman, P. D. Close to the edge: heterochromatin at the nucleolar and nuclear peripheries. *Biochim. Biophys. Acta Gene Regul. Mech.* **1864**, 194666 (2021). 33307247 10.1016/j.bbagrm.2020.194666 PMC7855492

[63] Briand, N. & Collas, P. Lamina-associated domains: peripheral matters and internal affairs. *Genome Biol.* **21**, 85 (2020). 32241294 10.1186/s13059-020-02003-5 PMC7114793

[64] Almassalha, L. M. et al. Chromatin conformation, gene transcription, and nucleosome remodeling as an emergent system. *Sci. Adv.* **11**, eadq6652 (2025). 39792661 10.1126/sciadv.adq6652 PMC11721585

[65] Shim, A. R. et al. Chromatin as self-returning walks: From population to single cell and back. *Biophys. Rep.* **2**, 100042 (2022). 10.1016/j.bpr.2021.100042 PMC9680733 36425085

[66] Carignano, M. A. et al. Local volume concentration, packing domains, and scaling properties of chromatin. *eLife* **13**, RP97604 (2024). 39331520 10.7554/eLife.97604 PMC11434620

[67] Kirmes, I. et al. A transient ischemic environment induces reversible compaction of chromatin. *Genome Biol.* **16**, 246 (2015). 26541514 10.1186/s13059-015-0802-2 PMC4635527

[68] Alisafaei, F. et al. Regulation of nuclear architecture, mechanics, and nucleocytoplasmic shuttling of epigenetic factors by cell geometric constraints. *Proc. Natl. Acad. Sci. USA* **116**, 13200–13209 (2019). 31209017 10.1073/pnas.1902035116 PMC6613080

[69] Damodaran, K. et al. Compressive force induces reversible chromatin condensation and cell geometry-dependent transcriptional response. *Mol. Biol. Cell* **29**, 3039–3051 (2018). 30256731 10.1091/mbc.E18-04-0256 PMC6333178

[70] Garate, X. et al. The relationship between nanoscale genome organization and gene expression in mouse embryonic stem cells during pluripotency transition. *Nucleic Acids Res.* **52**, 8146–8164 (2024). 38850157 10.1093/nar/gkae476 PMC11317139

[71] Martinez-Sarmiento, J. A., Cosma, M. P. & Lakadamyali, M. Dissecting gene activation and chromatin remodeling dynamics in single human cells undergoing reprogramming. *Cell Rep.* **43**, 114170 (2024). 38700983 10.1016/j.celrep.2024.114170 PMC11195307

[72] Wang, Y. et al. Chromatin organization governs transcriptional response and plasticity of cancer stem cells. *Adv. Sci.* **12**, 2407426 (2025). 10.1002/advs.202407426 PMC12061297 40051293

[73] Xu, J. et al. Ultrastructural visualization of chromatin in cancer pathogenesis using a simple small-molecule fluorescent probe. *Sci. Adv.* **8**, eabm8293 (2022). 35245126 10.1126/sciadv.abm8293 PMC8896800

[74] Xu, J. et al. Super-resolution imaging reveals the evolution of higher-order chromatin folding in early carcinogenesis. *Nat. Commun.* **11**, 1899 (2020). 32313005 10.1038/s41467-020-15718-7 PMC7171144

[75] Perez-Gonzalez, A., Bevant, K. & Blanpain, C. Cancer cell plasticity during tumor progression, metastasis and response to therapy. *Nat. Cancer* **4**, 1063–1082 (2023). 37537300 10.1038/s43018-023-00595-y PMC7615147

[76] Paul, M. R., Hochwagen, A. & Ercan, S. Condensin action and compaction. *Curr. Genet* **65**, 407–415 (2019). 30361853 10.1007/s00294-018-0899-4 PMC6421088

[77] Bloom, K. & Kolbin, D. Mechanisms of DNA mobilization and sequestration. *Genes* **13**, 352 (2022). 10.3390/genes13020352 PMC8872102 35205396

[78] Gabriele, M. et al. Dynamics of CTCF- and cohesin-mediated chromatin looping revealed by live-cell imaging. *Science* **376**, 496–501 (2022). 35420890 10.1126/science.abn6583 PMC9069445

[79] Nagano, T. et al. Single-cell Hi-C reveals cell-to-cell variability in chromosome structure. *Nature* **502**, 59–64 (2013). 24067610 10.1038/nature12593 PMC3869051

[80] Li, H. et al. Mapping chromatin structure at base-pair resolution unveils a unified model of cis-regulatory element interactions. *Cell* **188**, 7175–7193 e19 (2025). 41197626 10.1016/j.cell.2025.10.013 PMC7618578

[81] Harris, H. L. et al. Chromatin alternates between A and B compartments at kilobase scale for subgenic organization. *Nat. Commun.* **14**, 3303 (2023). 37280210 10.1038/s41467-023-38429-1 PMC10244318

[82] Albiez, H. et al. Chromatin domains and the interchromatin compartment form structurally defined and functionally interacting nuclear networks. *Chromosome Res.* **14**, 707–733 (2006). 17115328 10.1007/s10577-006-1086-x

[83] Williams, J. F. et al. The condensation of HP1alpha/Swi6 imparts nuclear stiffness. *Cell Rep.* **43**, 114373 (2024). 38900638 10.1016/j.celrep.2024.114373 PMC11348953

[84] Amar, K. et al. Effects of forces on chromatin. *APL Bioeng.* **5**, 041503 (2021). 34661040 10.1063/5.0065302 PMC8516479

[85] Spill, F. et al. Impact of the physical microenvironment on tumor progression and metastasis. *Curr. Opin. Biotechnol.* **40**, 41–48 (2016). 26938687 10.1016/j.copbio.2016.02.007 PMC4975620

[86] Miroshnikova, Y. A. & Wickstrom, S. A. Mechanical forces in nuclear organization. *Cold Spring Harb. Perspect Biol*. **14**, a039685 (2022). 10.1101/cshperspect.a039685 PMC8725626 34187806

[87] Vidi, P. A. et al. Closing the loops: chromatin loop dynamics after DNA damage. *Nucleus* **16**, 2438633 (2025). 39720924 10.1080/19491034.2024.2438633 PMC12897541

[88] Chatterjee, N. & Walker, G. C. Mechanisms of DNA damage, repair, and mutagenesis. *Environ. Mol. Mutagen* **58**, 235–263 (2017). 28485537 10.1002/em.22087 PMC5474181

[89] Das, R. et al. How enzymatic activity is involved in chromatin organization. *eLife* 11, e79901 (2022). 10.7554/eLife.79901 PMC9810329 36472500

[90] Pommier, Y. et al. Human topoisomerases and their roles in genome stability and organization. *Nat. Rev. Mol. Cell Biol.* **23**, 407–427 (2022). 35228717 10.1038/s41580-022-00452-3 PMC8883456

[91] Ochs, F. et al. Stabilization of chromatin topology safeguards genome integrity. *Nature* **574**, 571–574 (2019). 31645724 10.1038/s41586-019-1659-4

[92] Espinosa-Martinez, M., Alcazar-Fabra, M. & Landeira, D. The molecular basis of cell memory in mammals: the epigenetic cycle. *Sci. Adv.* **10**, eadl3188 (2024). 38416817 10.1126/sciadv.adl3188 PMC10901381

[93] Carter, B. & Zhao, K. The epigenetic basis of cellular heterogeneity. *Nat. Rev. Genet.* **22**, 235–250 (2021). 33244170 10.1038/s41576-020-00300-0 PMC10880028

[94] Dupont, S. & Wickstrom, S. A. Mechanical regulation of chromatin and transcription. *Nat. Rev. Genet.* **23**, 624–643 (2022). 35606569 10.1038/s41576-022-00493-6

[95] Schultz, E. R. et al. Current advances in genome modeling across length scales. *Wiley Interdiscip. Rev. Comput. Mol. Sci.* **15**, e70024 (2025).

[96] Frederick, J. et al. Leveraging chromatin packing domains to target chemoevasion in vivo. *Proc. Natl. Acad. Sci. USA* **122**, e2425319122 (2025). 40694328 10.1073/pnas.2425319122 PMC12318189

[97] Wang, B. et al. The molecular basis of lamin-specific chromatin interactions. *Nat. Struct. Mol. Biol.* **32**, 1999–2011 (2025). 40750945 10.1038/s41594-025-01622-5 PMC12527912

[98] Kreysing, J. P. et al. Molecular architecture of heterochromatin at the nuclear periphery of primary human cells. bioRxiv 10.1101/2025.04.09.647790 (2025). 10.1038/s41467-026-75087-5 PMC13332202 42399220

[99] Nunez, J. K. et al. Genome-wide programmable transcriptional memory by CRISPR-based epigenome editing. *Cell* **184**, 2503–2519 e17 (2021). 33838111 10.1016/j.cell.2021.03.025 PMC8376083

[100] Carnevali, D. et al. A deep learning method that identifies cellular heterogeneity using nanoscale nuclear features. *Nat. Mach. Intell.* **6**, 1021–1033 (2024). 39309215 10.1038/s42256-024-00883-x PMC11415298

[101] Vinayak, V. et al. Mollifier layers: Enabling efficient high-order derivatives in inverse PDE learning.* Transactions on Machine Learning Research (TMLR)* [Online] Available: https://openreview.net/forum?id=6mFVZSzyev (2025).

[102] Kim, H. H. et al. O-SNAP: a comprehensive pipeline for spatial profiling of chromatin architecture. bioRxiv 10.1101/2025.07.18.665612 (2025).

[103] Betzig, E. et al. Imaging intracellular fluorescent proteins at nanometer resolution. *Science* **313**, 1642–1645 (2006). 16902090 10.1126/science.1127344

[104] Schnitzbauer, J. et al. Super-resolution microscopy with DNA-PAINT. *Nat. Protoc.* **12**, 1198–1228 (2017). 28518172 10.1038/nprot.2017.024

[105] Eltsov, M. et al. Analysis of cryo-electron microscopy images does not support the existence of 30-nm chromatin fibers in mitotic chromosomes in situ. *Proc. Natl. Acad. Sci. USA* **105**, 19732–19737 (2008). 19064912 10.1073/pnas.0810057105 PMC2604964

